# Maternal genetic contribution to pre-pregnancy obesity, gestational weight gain, and gestational diabetes mellitus

**DOI:** 10.1186/s13098-019-0434-x

**Published:** 2019-05-14

**Authors:** Selvihan Beysel, Nilnur Eyerci, Mustafa Ulubay, Mustafa Caliskan, Muhammed Kizilgul, Merve Hafızoğlu, Erman Cakal

**Affiliations:** 1 Department of Endocrinology and Metabolism, Ankara Diskapi Yildirim Beyazit Teaching and Training Research Hospital, Ankara, Turkey; 2 Department of Medical Biology, Baskent University, Ankara, Turkey; 3 Department of Genetic Research, Ankara Diskapi Yildirim Beyazit Teaching and Training Research Hospital, Ankara, Turkey; 4 Department of Obstetrics and Gynecology, Gulhane School of Medicine, Ankara, Turkey; 5 Department of İnternal Medicine, Afyonkarahisar Saglik Bilimleri University, Afyon, Turkey; 6 Department of Endocrinology and Metabolism, Afyonkarahisar Saglik Bilimleri University, Afyon, Turkey

**Keywords:** Gestational weight gain, Polymorphisms, Gestational diabetes, Pre-pregnancy obesity

## Abstract

**Introduction:**

Pre-pregnancy obesity, gestational diabetes mellitus (GDM), and gestational weight gain (GWG) are associated with each other. This is the first study to investigate whether genetic variants were associated with having GDM, and whether genetic variants-related GDM were associated with adiposity including pre-pregnancy obesity and excessive GWG in Turkish women.

**Patients and methods:**

Women with GDM (n = 160) and without GDM (n = 145) were included in case-controlled study. Genotyping of the *HNF1A* gene (p.I27L rs1169288, p.98V rs1800574, p.S487N rs2464196), the *VDR* gene (p.BsmI rs1544410, p.ApaI rs7975232, p.TaqI rs731236, p.FokI rs2228570), and *FTO* gene (rs9939609) SNPs were performed by using RT-PCR.

**Results:**

The *FTO* AA genotype was associated with an increased risk of having GDM (AA vs. AT + TT, 24.4% vs. 12.4%, OR = 2.27, 95% CI [1.23–4.19], p = 0.007). The *HNF1A* p.I27L GT/TT genotype was associated with increased GDM risk (GT + TT vs. GG-wild, 79.4% vs. 65.5%, OR = 2.02, 95% CI 1.21–3.38], p = 0.007). However, all *VDR* gene SNPs and the *HNF1A* p.A98V, p.S487N were not associated with having GDM (p > 0.05). The *FTO* AA genotype was associated with an increased risk for pre-pregnancy overweight/obesity (OR = 1.43, 95% CI [1.25–3.4], p = 0.035), but not associated with excessive GWG after adjusting for pre-pregnancy weight (p > 0.05). Pre-pregnancy weight, weight at delivery, and GWG did not differ in both *VDR* and *HNF1A* gene carriers (p > 0.05). HOMA-IR and HbA1c were increased in both p.I27L TT and *FTO* AA genotype carriers (p < 0.05).

**Conclusion:**

The adiposity-related gene *FTO* is associated with GDM by the effect of *FTO* on pre-pregnancy obesity. The diabetes-related p.I27L gene is associated with GDM by increasing insulin resistance.

## Introduction

Maternal obesity and gestational diabetes mellitus (GDM) is a growing public health problem worldwide [1]. The Institute of Medicine (IOM) developed guidelines for gestational weight gain (GWG) during pregnancy; however, no specific recommendations could be made for GDM and multiethnic differences [2, 3]. Both pre-pregnancy obesity and excessive GWG are related to increased risk of maternal obesity and GDM [3]. Becoming pregnant or gaining too much weight during pregnancy are the risk factors for adverse perinatal complications and increased risk for future metabolic disease in overweight/obese women, both for the mothers and their offspring [1, 4]. Pre-pregnancy obesity and excessive GWG may have additive negative impact on maternal and neonatal outcomes in women with GDM [5, 6]. Pre-pregnancy obesity, gestational diabetes, and excessive GWG are associated with multiple factors such as the environment, behavior, and genetics; however, understanding these associations is complex [1, 3]. Diabetes-related or maternal and/or fetal adiposity-related genetic variants have been associated with GDM, pre-pregnancy weight, and GWG during pregnancy [7–9]. Kawai et al. reported that common type 2 diabetes risk variants were associated with increased risk of GDM [8]. Genetic variants were associated with GDM and progression to pre-diabetes and type 2 diabetes mellitus in women with prior GDM [9]. Evidence has been presented for a genetic predisposition to GDM risk and also a change in GWG during pregnancy [7, 10–13], and gene–environment interactions could explain the variation in GWG and GDM.

The fat mass and obesity-associated gene (*FTO*) rs9939609 single nucleotide polymorphism (SNP) was associated with increased risk of obesity and type 2 diabetes, as well as GDM [10]. The *FTO* SNPs have been reported to be associated with pre-pregnancy obesity [8] and excessive GWG [11]. The *FTO* variants related to type 2 diabetes are mediated by the effect of the FTO gene on body mass index (BMI); however, the exact mechanisms of this relation have not been identified [10, 11]. Vitamin D shows its cellular activity by binding to vitamin D receptors (VDR). VDR, as a transcription factor, has a role in the regulation of insulin secretion from pancreatic beta cells [14]. VDR has effect on proliferation, differentiation, and activation of immune cells and cytokine production, and subsequently type 2 diabetes occurs [15, 16]. Hepatocyte nuclear factor 1A (HNF1A), as a transcription factor, has a role in the function of pancreas beta cells [17]. Endocrine and exocrine pancreatic cells express HNF1A in the developmental stage. HNF1A is necessary for the glucose response to insulin secretion and glucose metabolism [18]. Women with *HNF1A* mutation are diagnosed as having monogenic form of diabetes type 3 (MODY3), and these women usually present with GDM, and diabetes persisting after delivery [17–19].

This is the first study to investigate the effect of *HNF1A* gene, *VDR* gene, and *FTO* gene variants on having GDM, pre-pregnancy obesity, and excessive GWG in Turkey. We aimed to examine whether these genetic variants would associate with having GDM, and then, whether the genetic variants that associated with GDM would associate with adiposity including pre-pregnancy obesity and excessive GWG. The *VDR* gene (encoding as SNPs p.BsmI, p.ApaI, p.TaqI, and p.FokI), and, *HNF1A* gene (encoding as SNPs p.I27L, p.A98V, and p.S487N) were chosen because these genetic variants have been reported to be associated with type 2 diabetes, as well as GDM risk [12, 14–20]. We also investigated the obesity-related *FTO* gene rs9939609 SNP because it is associated with both GDM and gestational body weight during pregnancy [10, 13, 20]. Genetic variants are implicated in the pathogenesis of GDM. Evidence suggests the genetic alterations in genes responsible for metabolic changes during pregnancy predispose to GDM [7]. We also hypothesized that these diabetes and adiposity-related genetic variants would likely be associated with GDM risk and gestational body weight during pregnancy.

## Patients and methods

### Study population

Pregnant women referred to tertiary hospital, Obstetrics and Gynecology Clinic, Ankara, from 2015 to 2016, were included in this case-control study. Women with GDM (n = 160) and age- and gestational age-matched women without GDM as controls (n = 145) were included in the study. Gestational age was assessed from the date of the last menstrual period and clinical assessment. A 2-h, 75-g oral glucose tolerance test at 24 to 28 weeks gestation age was performed for all pregnant women, irrespective of family history of DM or any other risk factors for GDM. Glucose concentrations after fasting, and 1 and 2 h after glucose administration < 92 mg/dl, < 180 mg/dl, and < 153 mg/dl, respectively, were considered normal. When the pregnant women’s glucose concentration was higher than any of these values, the women were diagnosed as having GDM [12]. Women whose GDM was diagnosed according to these criteria, aged 22–38 years, and whose pregnancy age was 24–48 weeks were included in the study. Women with GDM who had pre-existing type 2 diabetes, GDM observed in prior pregnancy, GDM with chronic disease such as hypertension, thyroid disorders, cardiac, hepatic or renal dysfunction were excluded. Women aged 22–38 years and with pregnancy age 24–28 weeks, with no GDM, type 2 diabetes, hypertension, thyroid disorders, cardiac, hepatic or renal dysfunction were accepted as controls and included in the study. Treatment of diet with or without insulin therapy was recorded. Weight, height, and systolic (SBP) and diastolic blood pressure (DBP) were measured in all participants. Body mass index (BMI, kg/m^2^) was calculated as weight (kg)/height^2^ (m^2^). Women were categorized as underweight (BMI < 18.5 kg/m^2^), normal weight (BMI = 18.5–24.9 kg/m^2^), overweight (BMI = 25–29.9 kg/m^2^), and obese (BMI ≥ 30 kg/m^2^). Maternal weight before pregnancy, pre-pregnancy weight, was obtained through a questionnaire. Maternal weight was measured at delivery. Gestational weight gain (GWG) was calculated as the difference between the maternal weight at delivery and pre-pregnancy weight. The recommended GWG was calculated based on IOM guidelines related with pre-pregnancy BMI: underweight, a gain of 12.5–18 kg; normal weight, a gain of 11.5–16 kg; overweight, a gain of 7–11.5 kg; and obese, a gain of 5–9 kg. After this, GWG was divided into three categories: low, if the weight was below the recommendation; adequate, if the weight gain was within the recommendation; and high, if the weight gain was above the recommendation [21]. Serum glucose, insulin, and glycated hemoglobin (HbA_1c_) concentrations were measured at 24–28 weeks of pregnancy. Insulin resistance was calculated using the homeostasis model assessment-insulin resistance (HOMA-IR): [fasting plasma insulin (µIU/ml) × fasting plasma glucose (mg/dl)]/405 [12]. This study was approval by Diskapi Yildirim Beyazit Teaching and Training Research Hospital Ethics Board (Number. 24.04.2015-13/25). Written informed consent was obtained from each participant.

### Genotyping

Genetic analyses for the *VDR* gene SNPs p.*FokI* (rs2228570), p.*BsmI* (rs1544410), p.*ApaI* (rs7975232), and p.*TaqI* (rs731236) and the *HNF1A* gene SNPs *p.S487N* (rs2464196, p.Ser486Asn), *p.A98V* (rs1800574, p.Ala98Val), *p.I27L* (rs1169288, p.Ile27Leu) and the *FTO* gene rs939609 SNPs were performed using real-time polymerase chain reaction (RT-PCR) amplification. Genomic DNA was isolated from collected peripheral blood samples of the subjects using DNA Isolation Kit (Roche Diagnostics, Indianapolis, IN, USA). Genotyping of each SNP in the *VDR* gene, *HNF1A* gene, and *FTO* gene was independently conducted using a pre-validated fluorescence-based allele-specific PCR assay, KASPar (KBiosciences, Hoddesdon, UK) and performed on a Rotor-Gene Q real-time cycler (Qiagen, Hilden, Germany) according to the manufacturer’s instruction. Allele discrimination was made using Rotor-Gene Q software *v.*2.3.1 (Qiagen, Hilden, Germany). The genotype calling was performed blind without information on the clinical phenotypes.

### Statistical analysis

Statistical analysis was performed using the SPSS 18.0 (SPSS, Inc) software. Variables are presented as mean ± standard deviation (SD) or median (min–max), percentages (%), odds ratios (OR), 95% confidence intervals (CI). Normality was tested using the Kolmogorov–Smirnov and Shapiro–Wilk *W* test. SNPs are expressed as allelic frequency (q) or prevalence of genotypes (%). Categorical variables were analyzed using the Chi-square test or Fisher’s exact test, where appropriate. Student’s *t*-test was used for normally distributed continuous variables or log-transformed variables between two groups. The Hardy–Weinberg equilibrium (HWE) at individual loci was assessed using the Chi-square test. Multiple logistic regression analysis and the Chi-square test or Fisher’s exact test was tested using models and ORs were calculated: dominant (major allele homozygotes vs. heterozygotes + minor allele homozygotes), recessive (major allele homozygotes + heterozygotes vs. minor allele homozygotes) and codominant (major allele homozygotes vs. heterozygote and minor allele homozygotes vs. major allele homozygotes). Pair-wise linkage disequilibrium (LD) and correlation coefficients (r^2^) were analyzed using the HAPLOVIEW program. We made a variable reflecting all possible combinations of genotypes for each SNP. Power analysis was performed using web-based software http://osse.bii.a-star.edu.sg/calculation2.php. The power of study was 65%. Statistical significance was defined as *p *< 0.05.

## Results

The mean age, gestational age, and height were similar between the women with GDM and controls (p > 0.05). Pre-pregnancy overweight/obesity were increased in women with GDM compared with controls (p < 0.05). Weight at delivery and excessive GWG were increased in women with GDM compared with the controls (p < 0.05). Serum glucose, insulin, HOMA-IR, and HbA1c were increased in women with GDM compared with the controls (p < 0.05, each). The clinical features of the subjects are shown in Table 1. Minor allele frequency of the *HNF1A*, *VDR*, and *FTO* genes is shown in Table 2. These frequencies were in HWE except p.A98V. Haploview analysis showed that the *HNF1A, VDR*, and *FTO* genes were not in LD. The risk alleles of the *HNF1A* gene (p.S487N, and p.A98V) and, *VDR* gene (p.ApaI, p.TaqI, p.BsmI and p.FokI) were similar between women with GDM and the controls (p > 0.05, each). Genotype analysis is shown in Table 3.

**Table 1:** Characteristics of subjects

	Controls (n = 145)	Gestational diabetes mellitus (n = 160)	p
Age (year)	28.25 ± 5.15	29.35 ± 5.36	0.075
Gestational age (weeks)	26.27 ± 1.48	25.99 ± 1.65	0.137
Height (cm)	160.40 ± 5.71	159.21 ± 5.95	0.076
Pre-pregnancy weight (kg)	61.74 ± 11.98	76.21 ± 11.27	*0.001*
Pre-pregnancy BMI (kg/m^2^)	24.06 ± 4.82	30.21 ± 5.10	*0.001*
Pre-pregnancy BMI (%)			*0.001*
Underweight (< 20 kg/m^2^)	23.4	3.8	
Normal weight (20–24.9 kg/m^2^)	38.6	8.8	
Overweight (25–29.9 kg/m^2^)	26.2	34.4	
Obesity (≥ 30 kg/m^2^)	11.7	53.1	
Pre-pregnancy overweight/obesity (%)^a^	37.9	87.5	*0.001*
Weight at delivery (kg)	77.60 ± 12.59	87.58 ± 11.54	*0.001*
BMI at delivery (kg/m^2^)	30.24 ± 5.18	34.71 ± 5.30	*0.001*
Gestational weight gain (kg)	16.05 ± 5.43	11.56 ± 2.72	*0.001*
Gestational weight gain (%)^b^			*0.011*
Excessive	44.1	61.2	
Adequate	46.9	33.1	
Below	9.0	5.6	
Glucose (mg/dl)	72.39 ± 7.12	101.67 ± 11.99	*0.001*
İnsulin (µIU/ml)	8.07 ± 2.02	11.93 ± 4.78	*0.001*
HOMA-IR	1.42 ± 0.39	3.06 ± 1.26	*0.001*
HbA1c (%)	5.01 ± 0.32	5.51 ± 0.43	*0.001*
Systolic BP (mmHg)	108.06 ± 8.74	110.84 ± 11.23	0.052
Diastolic BP (mmHg)	72.70 ± 5.62	73.48 ± 5.11	0.207

Italics represents significant p-values

*PPO* pre-pregnancy overweight/obesity, *GDM* gestational diabetes mellitus, *GWG* gestational weight gain, *BMI* body mass index, *BP* blood pressure, *HOMA-IR* homeostasis model assessment-insulin resistance, *HbA1c* hemoglobin A1c

^a^Prepregnancy overweight/obesity is defined as the percentage of subjects with having BMI ≥ 25 kg/m^2^

^b^Recommended gestational weight gain was calculated based on Institute of Medicine (IOM) recommendations according to pre-pregnancy BMI

**Table 2:** Minor allele frequency of polymorphisms

	Risk allele	MAF for study sample
HNF1A I27L rs1169288	T	0.44
HNF1A S487N rs2464196	T	0.37
HNF1A A98V rs1800574	T	0.10
VDR ApaI rs7975232	C	0.42
VDR TaqI rs731236	C	0.35
VDR BsmI rs1544410	G	0.45
VDR FokI rs2228570	T	0.35
FTO rs9939609	A	0.37

*MAF* minor allele frequency

**Table 3:** Genotype analysis of HNF1A gene, VDR gene and FTO gene polymorphisms

	Controls, n	Gestational diabetes, n	OR (95% CI)	p
FTO gene rs9939609 (%)				0.011*
Co-dominant wild type TT	73	59		
Heterozygous AT	54	62	1.42 (0.86–2.24)	0.169**
Homozygous AA	18	39	2.68 (1.39–4.13)	0.003***
Dominant (AT + AA/TT)	72 vs. 73	101 vs. 59	1.73 (1.12–2.74)	*0.018*
Recessive (AA/AT + TT)	18 vs. 127	39 vs. 121	2.27 (1.23–4.19)	*0.007*
HNF1 gene I27L rs1169288 (%)				0.009*
Co-dominant wild type GG	50	33		
Heterozygous GT	78	94	1.82 (1.13–3.12)	0.026**
Homozygous TT	17	33	2.94 (1.41–4.16)	0.003***
Dominant (GT + TT/GG)	95 vs. 50	127 vs. 33	2.02 (1.21–3.38)	*0.007*
Recessive (TT/GT + GG)	17 vs. 128	33 vs. 127	1.95 (1.13–3.49)	*0.036*
HNF1 gene S487N rs2464196 (%)				0.919*
Co-dominant wild type CC	61	64		
Heterozygous CT	62	72	1.10 (0.67–1.80)	0.684**
Homozygous TT	22	24	1.04 (0.52–2.04)	0.910***
Dominant (CT + TT/CC)	84 vs. 61	96 vs. 64	1.11 (0.70–1.76)	0.683
Recessive (TT/CT + CC)	22 vs. 123	24 vs 136	0.98 (0.52–1.84)	0.966
HNF1 gene A98V rs1800574 (%)				0.433*
Co-dominant wild type CC	121	130		
Heterozygous CT	22	24	1.01 (0.54–1.90)	0.962**
Homozygous TT	2	6	2.79 (0.55–12.45)	0.196***
Dominant model (CT + TT/CC)	24 vs. 121	30 vs. 130	1.16 (0.64–2.10)	0.615
Recessive model (TT/CT + CC)	2 vs. 143	6 vs. 154	2.78 (0.55–12.5)	0.196
VDR gene ApaI rs7975232 (%)				0.199*
Co-dominant wild type AA	52	48		
Heterozygous AC	73	78	1.15 (0.69–1.91)	0.571**
Homozygous CC	20	34	1.84 (0.93–3.62)	0.076***
Dominant (AC + CC/AA)	93 vs. 52	112 vs. 48	1.30 (0.80–2.10)	0.279
Recessive (CC/AA + AC)	20 vs. 125	34 vs. 126	1.68 (0.92–3.02)	0.088
VDR gene TaqI rs731236 (%)				0.472*
Co-dominant wild type TT	82	81		
Heterozygous CT	33	37	1.13 (0.64–1.98)	0.658**
Homozygous CC	30	42	1.41 (0.80–2.48)	0.222***
Dominant (CT + CC/TT)	63 vs. 82	79 vs. 81	1.26 (0.82–2.04)	0.301
Recessive (CC/CT + TT)	30 vs. 115	42 vs. 118	1.36 (0.81–2.32)	0.253
VDR gene BsmI rs1544410 (%)				0.461*
Co-dominant wild type AA	57	53		
Heterozygous AG	52	63	1.32 (0.78–2.24)	0.290**
Homozygous GG	36	45	1.37 (0.76–2.44)	0.284***
Dominant (AG + GG/AA)	88 vs. 57	108 vs. 53	1.34 (0.841–2.15)	0.215
Recessive (GG/AG + AA)	36 vs. 109	45 vs. 116	1.18 (0.71–1.97)	0.515
VDR gene FokI rs2228570 (%)				0.191*
Co-dominant wild type CC	78	76		
Heterozygous CT	43	44	1.05 (0.62–1.77)	0.855**
Homozygous TT	24	40	1.71 (0.94–3.10)	0.076***
Dominant (CT + TT/CC)	67 vs. 78	84 vs. 76	1.28 (0.82–2.01)	0.272
Recessive (TT/CT + CC)	24 vs. 121	40 vs. 120	1.68 (0.95–2.59)	0.070

Categorical variables were analyzed with Chi-square test or Fisher’s exact test, where appropriate. Multiple logistic regression analysis and Fisher’s exact test were tested using models: dominant (major allele homozygotes vs heterozygotes + minor allele homozygotes), recessive (major allele homozygotes + heterozygotes vs minor allele homozygotes) and codominant (major allele homozygotes vs heterozygote and minor allele homozygotes vs major allele homozygotes)

Italics represents significant p-values

*p Wild vs homozygous vs heterozygous

**p heterozygous vs wild

***p homozygous vs wild type

The *FTO* gene rs9939609 distribution was TT-wild, heterozygote AT, and homozygote AA at 50.3%, 37.2%, and 12.4% in the controls, and 36.9%, 38.8%, and 24.4% in women with GDM (p = 0.011). The *FTO* gene AA genotype was associated with an increased risk of GDM more than the TT/AT genotype in co-dominant, dominant, and recessive models (dominant: AT + AA vs. TT-wild, 63.1% vs. 49.7%, OR = 1.73, 95% CI [1.12–2.74], p = 0.018, and recessive: AA vs. AT + TT, 24.4 vs. 12.4%, OR = 2.27, 95% CI [1.23–4.19], p = 0.007) (Table 3). The *FTO* AA/AT genotype had a greater association with pre-pregnancy overweight/obesity than TT-wild genotype (p < 0.05) (Table 4). Pre-pregnancy weight (p < 0.05) and weight at delivery (p < 0.05) progressively increased from the AA genotype to the TT genotype. GWG was increased in AT/AA genotype compared with the TT genotype (p < 0.05). Serum glucose, insulin, HOMA-IR, and HbA1c were higher in the AA genotype compared with the TT genotype (p < 0.05). The *FTO* AA genotype was associated with a greater risk of pre-pregnancy overweight/obesity compared with AT/TT genotypes (OR = 1.43, 95% CI [1.25–3.4], p = 0.035). The *FTO* AA genotype was associated with excessive GWG risk compared with the TT and AT genotype (OR = 1.73, 95% CI [1.62–3.15], p = 0.034); however, this association was lost after adjusting for pre-pregnancy weight (OR = 1.1, 95% CI [0.94–2.38], p > 0.05).

**Table 4:** Clinics of pregnants according to the FTO gene rs9939609 SNP

	TT-wild (n = 132)	AT (n = 116)	AA (n = 57)	p*	p**	p***
Controls (%)	55.3 (n = 73)	46.6 (n = 54)	31.6 (n = 18)	0.169	*0.003*	0.060
Gestational diabetes mellitus (%)	44.7 (n = 59)	53.4 (n = 62)	68.4 (n = 39)			
Pre-pregnancy BMI (%)				*<* *0.001*	*0.001*	*0.011*
Underweight (< 20 kg/m^2^)	18.2	10.3	7.0			
Normal weight (20–24.9 kg/m^2^)	33.3	16.4	12.3			
Overweight (25–29.9 kg/m^2^)	19.7	44.8	26.3			
Obesity (≥ 30 kg/m^2^)	28.8	28.4	54.4			
Pre-pregnancy overweight/obesity (%)^a^	48.5 (n = 64)	73.3 (n = 85)	80.7 (n = 46)	*<* *0.001*	*0.001*	0.284
Gestational weight gain (%)^b^				*0.001*	*<* *0.001*	*0.014*
Below	12.1	3.4	3.6			
Adequate	51.5	37.9	16.1			
Excessive	36.4	58.6	80.4			
Excessive GWG (%)	36.4 (n = 48)	58.6 (n = 68)	80.4 (n = 46)	*0.001*	*<* *0.001*	*0.003*
Pre-pregnancy weight (kg)	65.79 ± 13.80	69.69 ± 11.31	76.78 ± 14.81	*0.016*	*<* *0.001*	*0.001*
Pre-pregnancy BMI (kg/m^2^)	25.80 ± 5.80	27.46 ± 5.03	30.36 ± 6.27	*0.017*	*<* *0.001*	*0.001*
Weight at delivery (kg)	78.52 ± 13.01	83.84 ± 9.80	90.78 ± 14.79	*0.001*	*<* *0.001*	*<* *0.001*
BMI at delivery (kg/m^2^)	30.77 ± 5.56	33.03 ± 4.72	35.86 ± 6.22	*0.001*	*<* *0.001*	*0.001*
Gestational weight gain (kg)	10.93 ± 3.77	12.93 ± 2.31	13.98 ± 4.91	*0.029*	*0.021*	0.654
Glucose (mg/dl)	84.64 ± 18.01	88.06 ± 17.65	91.64 ± 17.25	0.134	*0.014*	0.207
İnsulin (µIU/ml)	9.61 ± 4.35	10.18 ± 3.72	11.27 ± 4.89	0.315	*0.039*	0.148
HOMA-IR	2.16 ± 1.26	2.33 ± 1.18	2.65 ± 1.37	0.307	*0.033*	0.159
HbA1c (%)	5.22 ± 0.48	5.24 ± 0.41	5.41 ± 0.51	0.685	*0.018*	*0.027*
Systolic BP (mmHg)	110.41 ± 9.59	108.87 ± 10.61	108.77 ± 10.74	0.232	0.298	0.951
Diastolic BP (mmHg)	73.74 ± 5.43	72.81 ± 5.03	72.24 ± 5.75	0.169	0.090	0.503

Italics represents significant p-values

*PPO* pre-pregnancy overweight/obesity, *GDM* gestational diabetes mellitus, *GWG* gestational weight gain, *BMI* body mass index, *BP* blood pressure, *HOMA-IR* homeostasis model assessment-insulin resistance, *HbA1c* hemoglobin A1c

*p TT wild type vs heterozygote AT

**p TT wild type vs homozygote AA

***p heterozygote AT vs homozygote AA

^a^Prepregnancy overweight/obesity is defined as the percentage of subjects with having BMI ≥ 25 kg/m^2^

^b^Recommended gestational weight gain was calculated based on Institute of Medicine (IOM) recommendations according to pre-pregnancy BMI

The *HNF1A* gene p.I27L distribution of GG-wild, GT, and TT was 34.5%, 53.8%, and 11.7% in the controls, and 20.6%, 58.8%, and 20.6% in women with GDM (p = 0.009). The *HNF1A* gene p.I27L TT/GT genotype was associated with a greater risk of GDM in comparison with the GG genotype in co-dominant, dominant, and recessive models (dominant: GT + TT vs. GG-wild, 79.4 vs. 65.5%, OR = 2.02, 95% CI [1.21–3.38], p = 0.007 and recessive: TT vs. GT + GG, 20.6 vs. 11.7%, OR = 1.95, 95% CI [1.13–3.49], p = 0.036) (Table 3). Pre-pregnancy weight, weight at delivery, and GWG were similar between p.I27L genotypes (p > 0.05) (Table 5). Glucose, HOMA-IR, and HbA1c were increased in the p.I27L TT genotype compared with the GG-wild type (p < 0.05). Pre-pregnancy weight, weight at delivery, and GWG did not differ between the *VDR* and *HNF1A* gene carriers (p > 0.05).

**Table 5:** Clinics of pregnant women according to the HNF1A gene p.I27L

	GG wild (n = 83)	GT (n = 172)	TT (n = 50)	p*	p**	p***
Controls (%)	60.2 (n = 50)	45.3 (n = 78)	34.0 (n = 17)	*0.026*	*0.003*	0.153
Gestational diabetes mellitus (%)	39.8 (n = 33)	54.7 (n = 94)	66.0 (n = 33)			
Pre-pregnancy BMI (%)				0.653	0.622	0.695
Underweight (< 20 kg/m^2^)	15.7	13.4	8.0			
Normal weight (20–24.9 kg/m^2^)	21.7	23.3	24.0			
Overweight (25–29.9 kg/m^2^)	33.7	27.9	34.0			
Obesity (≥ 30 kg/m^2^)	28.9	35.5	34.0			
Pre-pregnancy overweight/obesity (%)^a^	62.7 (n = 52)	63.4 (n = 109)	68.0 (n = 34)	0.911	0.532	0.547
Gestational weight gain (%)^b^				0.112	0.804	0.342
Below	3.6	9.4	6.0			
Adequate	45.8	35.1	46.0			
Excessive	50.6	55.6	48.0			
Excessive GWG (%)	50.6 (n = 42)	55.8 (n = 96)	48.0 (n = 24)	0.434	0.771	0.329
Pre-pregnancy weight (kg)	67.93 ± 13.64	70.12 ± 14.28	68.94 ± 11.42	0.247	0.665	0.592
Pre-pregnancy BMI (kg/m^2^)	26.78 ± 5.74	27.51 ± 6.06	27.35 ± 5.24	0.364	0.568	0.870
Weight at delivery (kg)	81.84 ± 13.34	83.56 ± 13.50	81.98 ± 10.73	0.338	0.951	0.445
BMI at delivery (kg/m^2^)	32.25 ± 5.66	32.77 ± 5.88	32.50 ± 5.13	0.503	0.795	0.772
Gestational weight gain (kg)	14.02 ± 4.60	13.67 ± 4.83	13.24 ± 4.98	0.583	0.359	0.434
Glucose (mg/dl)	83.89 ± 17.10	86.88 ± 17.69	94.06 ± 18.23	0.203	*0.002*	*0.013*
İnsulin (µIU/ml)	9.52 ± 3.16	10.30 ± 4.76	10.64 ± 4.09	0.215	0.108	0.681
HOMA-IR	2.10 ± 1.01	2.36 ± 1.37	2.54 ± 1.23	0.155	*0.045*	0.470
HbA1c (%)	5.15 ± 0.40	5.29 ± 0.50	5.32 ± 0.41	*0.048*	*0.037*	0.736
Systolic BP (mmHg)	109.93 ± 10.07	109.27 ± 10.26	109.70 ± 10.37	0.626	0.896	0.797
Diastolic BP (mmHg)	73.20 ± 5.47	73.11 ± 5.26	72.94 ± 5.63	0.901	0.790	0.838

Italics represents significant p-values

*PPO* pre-pregnancy overweight/obesity, *GDM* gestational diabetes mellitus, *GWG* gestational weight gain, *BMI* body mass index, *BP* blood pressure, *HOMA-IR* homeostasis model assessment-insulin resistance, *HbA1c* hemoglobin A1c

*p wild GG vs heterozygote GT

**p wild GG vs homozygote TT

***p heterozygote GT vs homozygote TT

^a^Prepregnancy overweight/obesity is defined as the percentage of subjects with having BMI ≥ 25 kg/m^2^

^b^Recommended GWG was calculated based on Institute of Medicine (IOM) recommendations according to pre-pregnancy BMI

## Discussion

Both the *FTO* AA genotype and *HNF1A* p.I27L GT/TT genotype were associated with an increased risk of having GDM in Turkish women. However, the *VDR* gene (p.ApaI, p.TaqI, p.FokI, p.BsmI) and *HNF1A* gene (p.A98V, p.S487N) were not associated with having GDM. Insulin resistance and impaired glucose metabolism was observed in both p.I27L TT and *FTO* AA genotype carriers. The *FTO* AA genotype was associated with an increased risk for pre-pregnancy overweight/obesity, but not associated with excessive GWG after adjusting for pre-pregnancy weight. The association of the adiposity-related gene FTO with GDM might be mediated by the effect of FTO on pre-pregnancy obesity. The diabetes-related p.I27L gene was associated with GDM by increasing insulin resistance.

Our results demonstrated that the *VDR* gene p.ApaI, p.TaqI, p.BsmI, and p.FokI genotypes were not associated with having GDM in Turkish women. The *VDR* gene and *HNF1A* gene SNPs were not associated with pre-pregnancy weight, weight at delivery, and GWG during pregnancy. The associations of the *VDR* gene and *HNF1A* gene with pre-pregnancy weight, weight at delivery, and GWG have not been investigated in previous studies. El-Beshbishy et al. reported that p.BsmI and p.FokI were not associated with GDM in Saudi women [22]. Incompatible to our results, p.FokI [23], p.ApaI, and p.TaqI [22] were associated with an increased risk of GDM in Iranian women [24]. We found that the *HNF1A* gene p.A98V and p.S487N were not associated with GDM in Turkish women. Zurawek et al. reported that p.I27L, p.A98V, and p.S487N were not associated with GDM in Polish women [25]. No relationship was reported between p.A98V and GDM in Danish women [12]; however, insulin secretion was decreased in p.A98V carriers without GDM [26], which is compensated by increasing insulin sensitivity [27]. Our data show that the *HNF1A* gene p.I27L GT/GG genotype was associated with an increased risk of GDM (OR = 2.02, 95% CI [1.21–3.38], p = 0.007). Pre-pregnancy weight, weight at delivery, and GWG were not associated with p.I27L genotypes. Insulin resistance and impaired glucose metabolism was observed in p.I27L TT carriers. We suggest that the diabetes-related p.I27L gene was associated with the increased risk of GDM by impairing glucose metabolism and increasing insulin resistance. Similarly, p.I27L was associated with an increased GDM risk in Scandinavian women by the effect of p.I27L on pancreas beta cell function [28] and insulin resistance [29]. Decreased beta cell function/transcriptional activity, decreased glucose-stimulated insulin secretion, increased insulin resistance, and increased type2 diabetes risk have been found in p.I27L + p.S487N carriers (if also including p.A98V) [27, 30, 31]. HNF1A controls beta cell function by regulating target genes such as glucose transporter 2, liver pyruvate kinase, collectrin, hepatocyte growth factor activator, and *HNF4A*. Decreased HNF1A activity causes decreased beta cell mass and expression of these target genes, which lead to impaired insulin secretion [17, 18]. Beta-cell dysfunction is more prone to developing impaired glucose tolerance during pregnancy [28].

The *FTO* gene AA genotype was associated with an increased risk of having GDM (OR = 2.27, 95% CI [1.23–4.19], p = 0.007). The *FTO* AA genotype had a greater risk for pre-pregnancy overweight/obesity (OR = 1.43, 95% CI [1.25–3.4], p = 0.035). The *FTO* AA genotype was not associated with GWG after adjusting for pre-pregnancy weight (OR = 1.1, 95% CI [0.94–2.38], p > 0.05). Insulin resistance and impaired glucose metabolism were observed in *FTO* AA genotype carriers. We suggest that the adiposity-related gene *FTO* was associated with increased risk of GDM by increasing pre-pregnancy obesity. Similarly, previous studies have shown that the *FTO* rs9939609 AA genotype was associated with higher pre-pregnancy weight [10, 13, 32]. Lawlor et al. reported that maternal fat or fetal fat adiposity-related variants were not associated with excessive GWG, but the *FTO* gene was associated with pre-pregnancy overweight [33]. The *FTO* gene has a role in the regulation of adiposity-related phenotypes through the effect of FTO on weight gain during younger ages [34] and continues throughout life [10]. FTO is expressed in the hypothalamic region, which regulates appetite [35], and this would contribute to energy intake and body fat mass [36]. Our data demonstrated that *FTO* gene AA genotype carriers were heavier before pregnancy, but AA carriers did not have significant weight gain during pregnancy. Chiou et al. reported that the *FTO* gene was associated with pre-pregnancy obesity and a tendency to gain less weight throughout pregnancy [5]. Consistent with our data, the *FTO* gene was not associated with greater GWG after adjusting for pre-pregnancy BMI in Caucasian and African-American populations [37]. The *FTO* gene was not associated with GWG according to the period of pregnancy in British [33] and Brazilian women [10]. Moreover, GWG comprises other factors such as the fetus, amniotic fluid, and placenta [10]. Pregnant women have biologic, behavioral, and hormonal changes throughout pregnancy [11]. Pre-pregnancy body weight shows maternal nutritional changes before conception, whereas GWG represents fetal-maternal physiologic conditions associated with genetic and nutrition factors [1]. This could modify the genetic contributions of the maternal *FTO*, *HNF1A*, and *VDR* gene variants on pre-gestational weight and GWG, as well as GDM [13, 33]; however it is not fully known which of these conditions is more associated with these disorders.

There are some limitations in our study that should be considered. We did not report the GWG according to gestational weeks. The small sample size resulted in a lower power for investigating a significant effect of any of the *HNF1A*, *VDR*, and *FTO* gene SNPs on weight changes during pregnancy. Also, we did not control our data for confounding variables such as nutrition, education, smoking and parity.

## Conclusion

Both the *FTO* AA genotype and *HNF1A* p.I27L GT/TT genotype were associated with increased GDM risk in Turkish pregnant women. However, the *VDR* gene p.ApaI, p.TaqI, p.FokI, p.BsmI and the *HNF1A* gene p.A98V, p.S487N genotypes were not associated with having GDM. The diabetes-related p.I27L gene was associated with GDM by increasing insulin resistance. The diabetes-related *HNF1A* p.I27L gene was associated with insulin resistance, which might contribute to developing GDM. The *FTO* AA genotype was associated with pre-pregnancy overweight/obesity, but did not contribute to significant weight gain during pregnancy. The adiposity-related gene *FTO* was associated with GDM by the effect of FTO on pre-pregnancy obesity. The *FTO* gene was associated with pre-pregnancy obesity, which might contribute to developing GDM. Genetic factors involved in GDM, pre-pregnancy weight, and GWG should be identified for the prevention of adverse complications of GDM and obesity during pregnancy. Further studies with multiethnic and larger populations are needed to find genetic variants related to GDM, pre-pregnancy obesity, and GWG during pregnancy.

## Data Availability

All data are freely available for scientific purpose.

## Abbreviations

GDM: gestational diabetes mellitus
GWG: gestational weight gain
BMI: body mass index
SNPs: single nucleotide polymorphisms
IOM: Institute of Medicine
HNF1A: hepatocyte nuclear factor 1α
FTO: the fat mass and obesity associated gene
VDR: vitamin D receptor
HOMA-IR: homeostasis model assessment-insulin resistance
HbA1c: hemoglobin A1c
